# Diffusion-weighted imaging in relation to morphology on dynamic contrast enhancement MRI: the diagnostic value of characterizing non-puerperal mastitis

**DOI:** 10.1007/s00330-017-5051-1

**Published:** 2017-09-27

**Authors:** Lina Zhang, Jiani Hu, Nicholas Guys, Jinli Meng, Jianguo Chu, Weisheng Zhang, Ailian Liu, Shaowu Wang, Qingwei Song

**Affiliations:** 1 Department of Radiology, First Affiliated Hospital of Dalian Medical University, 222 Zhongshan Road, Xigang, Dalian, Liaoning 116011 China; 2 Department of Radiology, Wayne State University, 540 East Canfield Street, Detroit, MI 48201 USA; 3 Department of Radiology, Chengban Branch of West China Hospital, 37 Guoxue Alley, Wuhou, Chengdu, Sichuan 610041 China; 4 Department of Radiology, Second Affiliated Hospital of Dalian Medical University, 467 Zhongshan Road, Shahekou, Dalian, Liaoning 116023 China

**Keywords:** mastitis, granulomatous mastitis, abscess, diffusion magnetic resonance imaging, image enhancement

## Abstract

**Objectives:**

To demonstrate the value of diffusion-weighted imaging (DWI) in the characterisation of mastitis lesions.

**Methods:**

Sixty-one non-puerperal patients with pathologically confirmed single benign mastitis lesions underwent preoperative examinations with conventional MRI and axial DWI. Patients were categorised into three groups: (1) periductal mastitis (PDM), (2) granulomatous lobular mastitis (GLM), and (3) infectious abscess (IAB). Apparent diffusion coefficient (ADC) values of each lesion were recorded. A one-way ANOVA with logistic analysis was performed to compare ADC values and other parameters. Discriminative abilities of DWI modalities were compared using the area under the receiver operating characteristic curve (AUC). *P* < 0.05 was considered statistically significant.

**Results:**

ADC values differed significantly among the three groups (*P* = 0.003) as well as between PDM and IAB and between PDM and GLM. The distribution of non-mass enhancement on dynamic contrast-enhanced (DCE) MRI differed significantly among the three groups (*P* = 0.03) but not between any two groups specifically. There were no differences in lesion location, patient age, T_2_WI or DWI signal intensity, enhancement type, non-mass internal enhancement, or mass enhancement characteristics among the three groups.

**Conclusions:**

ADC values and the distribution of non-mass enhancement are valuable in classifying mastitis subtypes.

***Key points*:**

*• Mastitis subtypes exhibit different characteristics on DWI and DCE MRI.*

*• ADC values are helpful in isolating PDM from other mastitis lesions.*

*• Distribution of non-mass enhancement also has value in comparing mastitis subtypes.*

## Introduction

Mastitis is primarily defined as infectious (usually bacterial) or non-infectious inflammation of breast tissue [1].. Non-puerperal mastitis describes inflammatory lesions of the breast that occur unrelated to pregnancy and breastfeeding [2]. Currently, non-puerperal mastitis accounts for approximately 4-5% of benign breast lesions, but its incidence is increasing, especially in developing countries [3]. For example, non-puerperal mastitis represents approximately 2-5% of all breast lesions in China, as compared to 0.3-1.9% of all breast lesions globally [3, 4].

Non-infectious non-puerperal mastitis is subclassified as periductal mastitis (PDM) or granulomatous lobular mastitis (GLM), while infectious non-puerperal mastitis most commonly manifests as an infectious abscess (IAB). The most common pathological characteristics of PDM, GLM, and IAB are as follows: PDM: greyish discharge exuding from dilated lactiferous ducts, resulting in plasma cell-induced inflammation; GLM: chronic, non-caseating granulomatous lobulitis; and IAB: accumulation of pus in a localised region of the breast [5].

Diffusion-weighted imaging (DWI) is an alternative, non-contrast functional magnetic resonance imaging (MRI) technique that is sensitive to the motion of free water molecules in biological tissues, which can reflect the histological characteristics of various lesions [9–12]. DWI often complements conventional MRI data in differentiating mastitis from various cancers and has potential value in classifying various cancer types as well [13–15]. The apparent diffusion coefficient (ADC), a quantitative parameter derived from DWI, has been shown to help differentiate between tumour subtypes [10, 16]. An anomalous variation in the ADC value may indicate changes such as oedema, cysts, bleeding, thick content, or necrosis [10, 13, 14].

However, to date, few DWI studies have focused specifically on mastitis lesions, and there currently exists in the literature a paucity of studies examining the DWI and dynamic contrast-enhanced (DCE) MRI characteristics of the different subtypes of mastitis [13, 14]. To ensure optimal patient outcomes, it is important to classify non-puerperal mastitis before administering therapies. For this reason, our study sought to determine the feasibility of using DWI combined with DCE MRI in evaluating PDM, GLM, and IAB. Utilising DWI and DCE MRI, our aim was to better characterise non-puerperal mastitis lesions and help reduce the interval between definitive diagnosis and administration of specific therapies.

## Materials and methods

### Patients and lesions

This retrospective study was performed from January 2010 to August 2015. Initially, seventy-five female patients underwent pathological assessments and MRI examinations for confirmation of non-puerperal mastitis. Fourteen of these 75 patients were excluded due to incomplete DCE or DWI sequences (as a result of an informatics failure in archiving image scans, including motion artefacts). Thus, 61 female patients with unilateral mastitis were enrolled in this study, assessed using DCE and DWI MRI scans, and categorised into three sub-groups based on the type of mastitis lesion (17 PDM: mean age = 44.47 ± 14.68; 32 GLM: mean age = 41.34 ± 12.71; 12 IAB: mean age = 41.33 ± 18.79). In 51 patients, a pathological diagnosis was made using percutaneous biopsies; in the other 10 patients, a pathological diagnosis was confirmed with surgery. All mastitis lesions were unilateral, and the majority occupied multiple quadrants within the affected breast. The mean age of clinical presentation for each group (i.e. PDM, GLM, and IAB) was consistent with previous reports [4, 17].

### Imaging protocols

All patients were imaged using a 3.0T whole-body MR scanner (Signa Excite HDxt, General Electric Healthcare, Milwaukee, WI, USA) with a dedicated eight-channel bilateral phase-array breast coil. Each patient was placed in prone position without compression. Conventional MR scanning sequences included sagittal fat-saturation FSE T_2_WI (repetition time/echo time [TR/TE] = 2600 ms/85 ms; slice thickness = 5 mm; number of excitations [NEX] = 2; matrix size = 288 × 224 zero-filled to 512× 256; field of view [FOV] = 20 cm × 20 cm) and axial short tau inversion recovery (STIR) sequence (TR/TE = 4450 ms/68 ms; inversion time [TI] = 210 ms; slice thickness = 5 mm; NEX = 1; matrix size = 256× 160 zero-filled to 256× 256; FOV = 40cm × 40 cm). DWI was acquired for both breasts in the axial plane with echo planar imaging (TR/TE = 8000 ms/90 ms; slice thickness = 3mm; NEX = 6; matrix size = 96×128 zero-filled to 128× 256; FOV = 30 cm × 30 cm; b = 0 and 800 s/mm^2^) before contrast agent injection. One pre- and seven post-contrast images were acquired using an axial 3D T1-weighted VIBRANT (Volume Imaging for Breast Assessment) sequence with fat saturation: TR/TE = 3.7 ms/2.1 ms; TI = 12 ms; flip angle [FA] = 10°; slice thickness = 2.2 mm; NEX = 1; matrix size = 320× 320 zero-filled to 512 × 512; FOV = 32 cm × 32 cm. Parallel imaging of auto-calibrating reconstruction for cartesian sampling (ARC) was employed. The first post-contrast acquisition was started 25 sec after contrast injection, and the remaining images were acquired every 57 sec. The contrast agent (Gadodiamide) was administrated with a dosage of 0.1 mmol/kg body weight and a mean volume of 15±3mL for each patient. Contrast was administered at a rate of 2.0 mL/s using a dedicated power injector.

### Imaging analysis

MRI results were interpreted independently by two radiologists, both with more than ten years of diagnostic experience in breast imaging. A diagnostic consensus was reached following a discussion of the images. All data was transferred to a GE workstation (Advantage Windows 4.5, General Electric, Madison, WI, USA). DCE morphology of each lesion was assessed in initial enhancement phases (90 s) according to the American College of Radiology (ACR) breast imaging-reporting and data system (BI-RADS) Breast MRI Lexicon (5^th^ edition, 2013) [18]. The region of interest (ROI) was obtained manually on the DWI with hyperintensity signal, ideally without necrotic or cystic components. When DWI could not clearly distinguish a mastitis lesion from a necrotic or cystic lesion (often because of its poor spatial resolution), DCE image was used to help to draw the ROI, which corresponding with the most enhanced part of the lesion on DCE. The mean value of three ROI measurements was obtained as the final ADC value.

### Statistical analysis

A one-way ANOVA and post-hoc SNK test were used for analysing ADC values among the three groups (PDM, GLM, and IAB) and between any two groups, respectively. The discriminative abilities of ADC values in arriving at a differential diagnosis were compared using receiver operating characteristic (ROC) curves and the area under the ROC curve (AUC). Conventional MRI and DCE MRI morphologies and clinical information, such as age of presentation and lesion location, were analysed using a chi-square test or Fisher’s exact test. Analysis was performed using SPSS software (SPSS Version 17, SPSS Inc., Chicago, IL, USA). *P* < 0.05 was considered indicative of a statistically significant difference among the groups. *P* < 0.0167 indicated a significant difference between any two groups compared using a chi-square test. Results of the SNK test did not provide *p*-values.

## Results

**Fig. 1:**
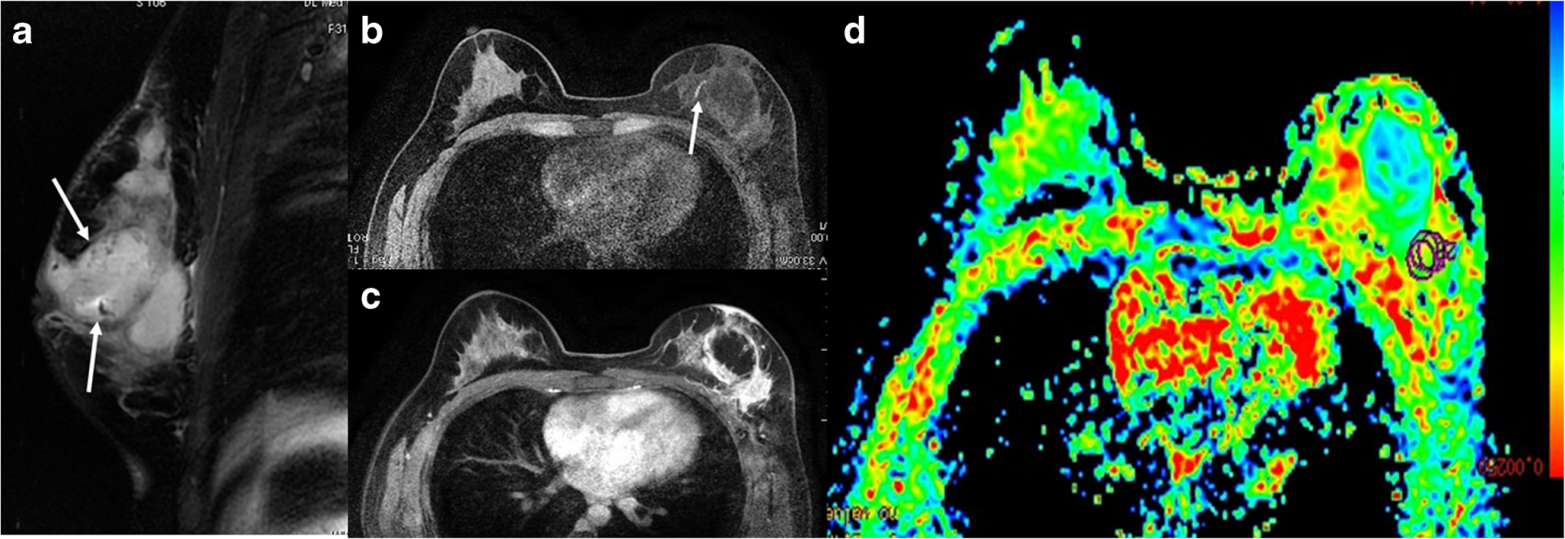
**(a-d):** MRI presentation of 31-year-old patient with left-breast PDM: a) Sagittal T_2_WI shows hyperintense signal of the lesion (arrow); b) Axial FSPGR shows linear hyperintense signal (indicative of duct ectasia, arrow) within the lesion; c) DCE MRI illustrates non-mass, multiple-region heterogeneous enhancement of the lesion; d) ADC map gives ADC value of 1.597 × 10^-3^ mm^2^/s (b = 800 s/mm^2^).

**Fig. 2:**
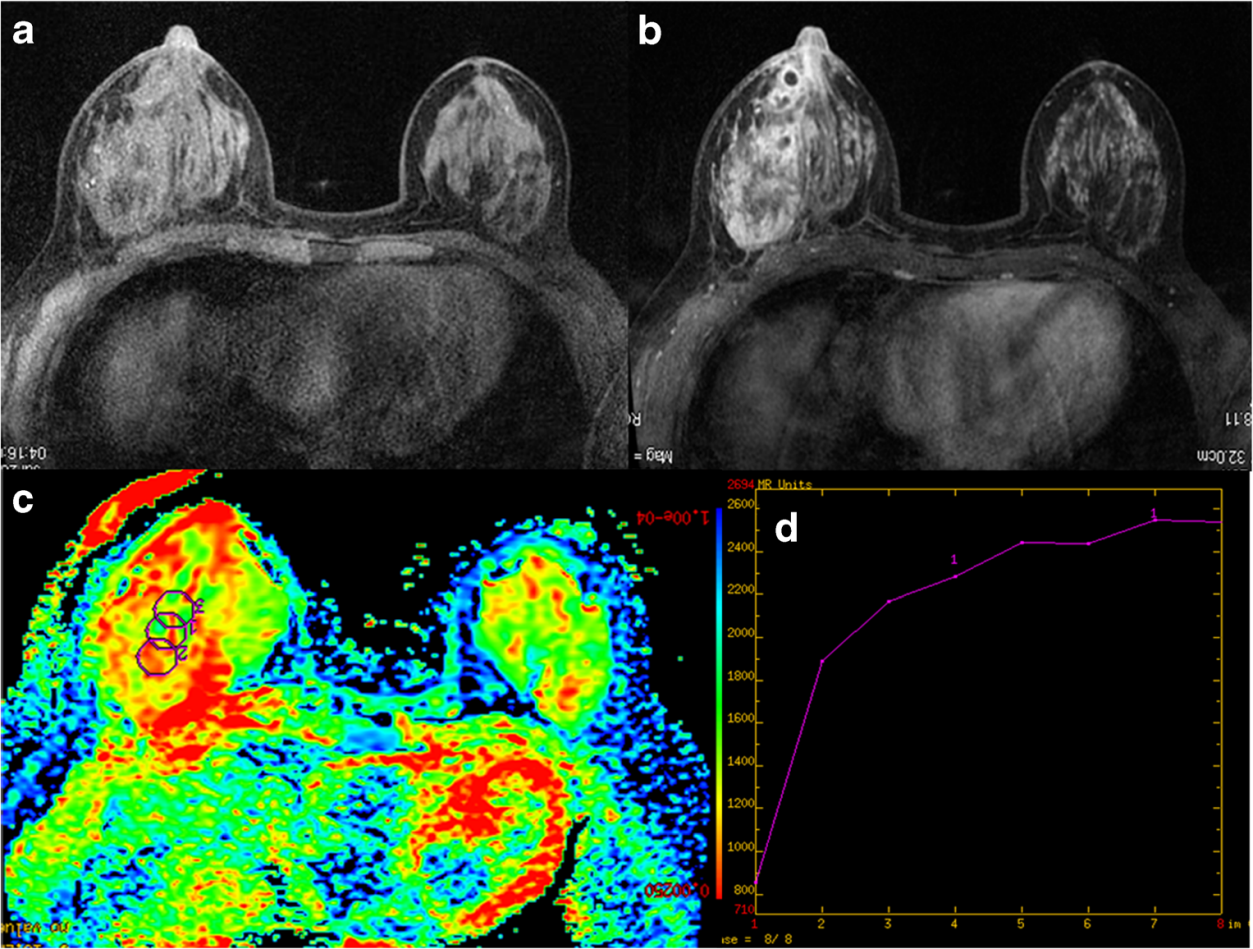
**(a-d):** MRI presentation of 43-year-old patient with right-breast PDM: a) Axial FSPGR shows hypointense signal of the lesion; b) DCE MRI illustrates non-mass, diffuse clustered ring enhancing lesion; c) ADC map gives ADC value of 1.87 × 10^-3^ mm^2^/s (b = 800 s/mm^2^); d) Time-signal intensity curve (TIC) shows persistent enhancement.

**Fig. 3:**
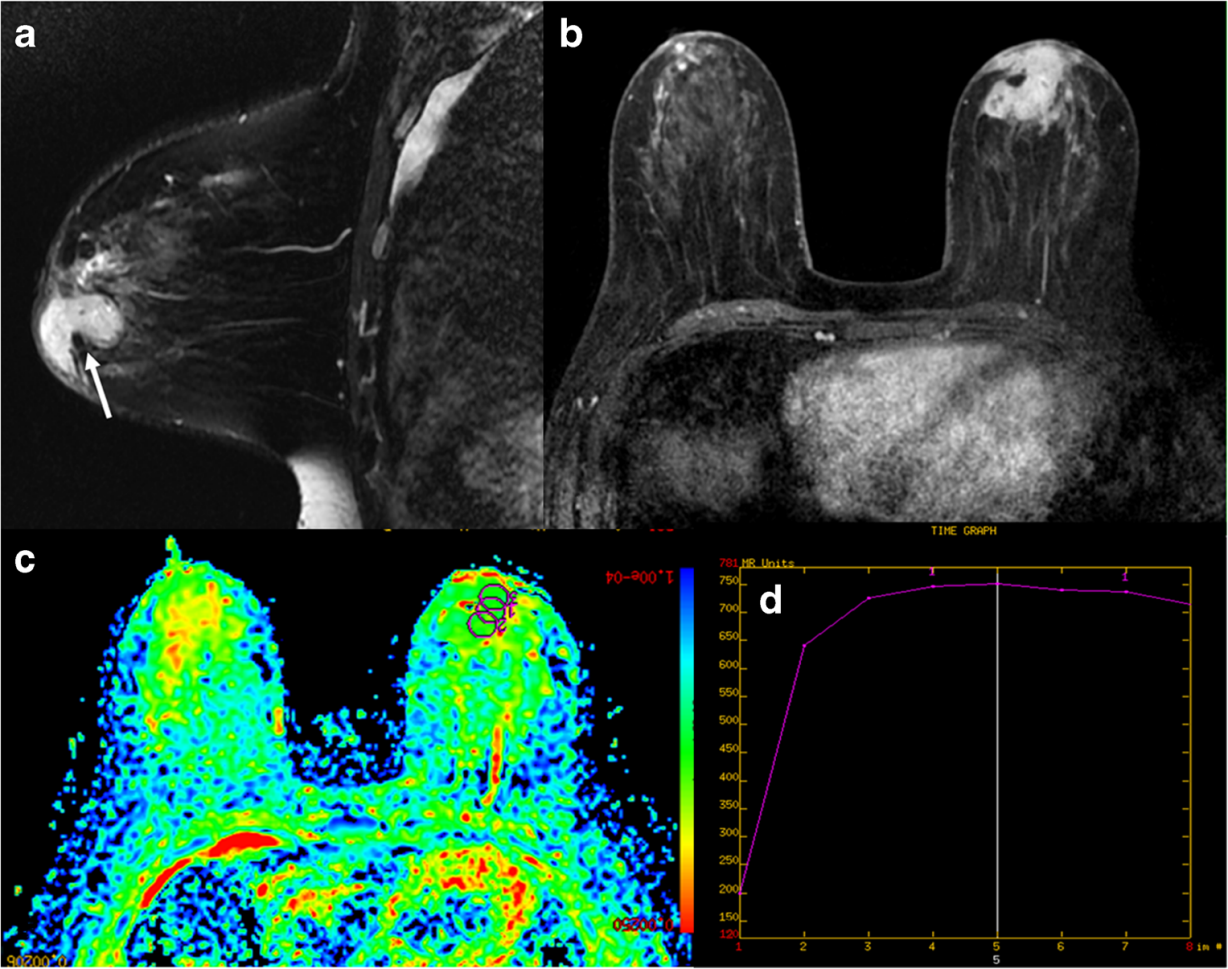
**(a-d):** MRI presentation of 41-year-old patient with left-breast GLM: a) Sagittal T_2_WI shows hyperintense signal of the lesion (arrow); b) DCE MRI illustrates mass enhancement of lesion with irregular shape, circumscribed margin, and heterogeneous internal enhancement; c) ADC map gives ADC value of 1.35 × 10^-3^ mm^2^/s (b = 800 s/mm^2^); d) Time-signal intensity curve (TIC) shows plateau enhancement.

**Fig. 4:**
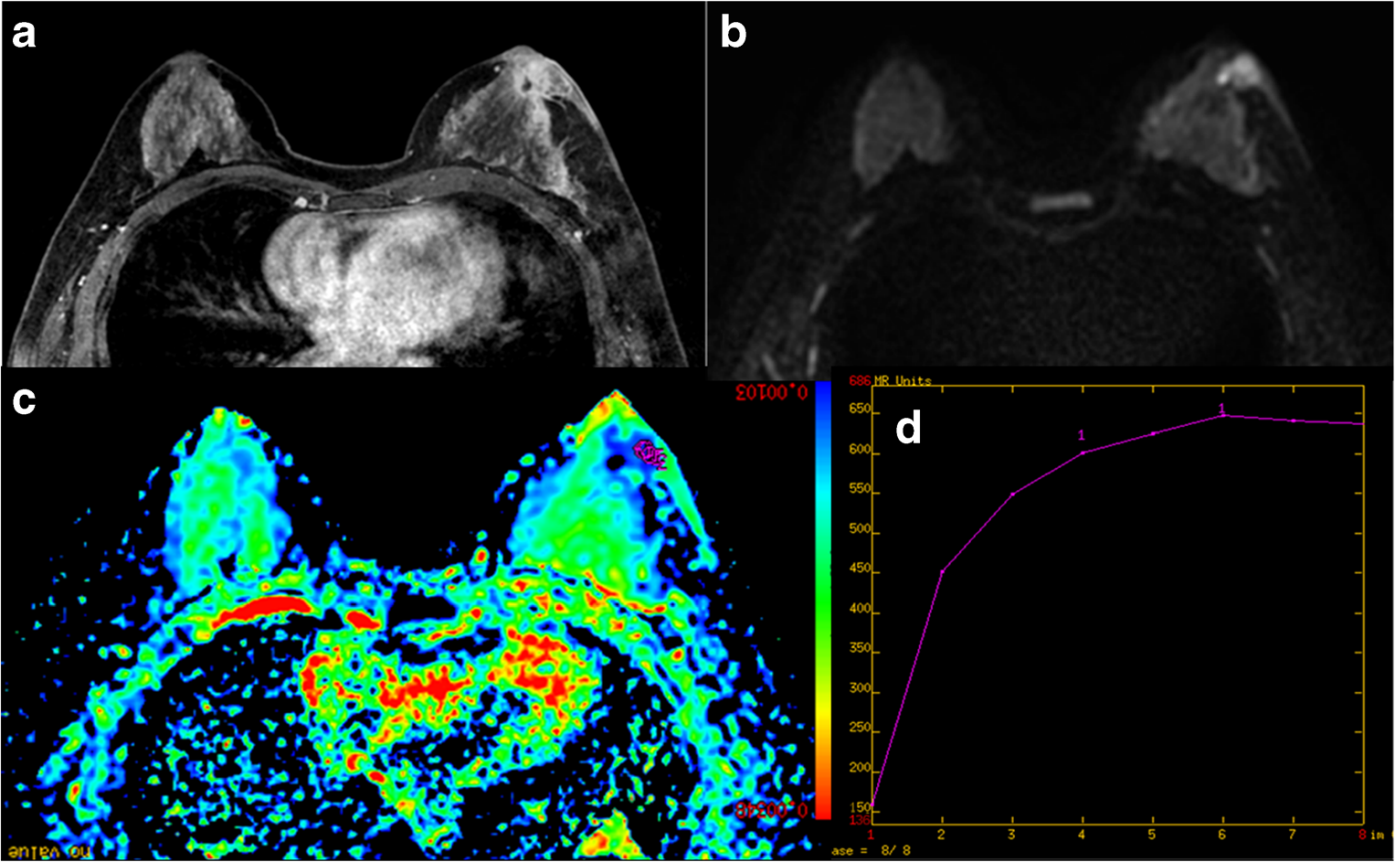
**(a-d):** MRI presentation of 42-year-old patient with right-breast IAB: a) DCE MRI illustrates non mass enhancement of lesion with regional and heterogeneous enhancement; b) DWI (b = 800 s/mm^2^) shows hyperintense signal of the lesion; c) ADC map gives ADC value of 1.09 × 10^-3^ mm^2^/s; d) Time-signal intensity curve (TIC) shows persistent enhancement.

The mean ADC values for the three groups were as follows: PDM: (1.558 ± 0.34) × 10^-3^ mm^2^/s; GLM: (1.244 ± 0.32) × 10^-3^ mm^2^/s; and IAB: (1.105 ± 0.44) × 10^-3^ mm^2^/s. One-way ANOVA revealed a significant difference in ADC values among the three groups (F = 6.589, *P* = 0.003). Post-hoc SNK test results are shown in Table 1. There was a significant difference between PDM and GLM and between PDM and IAB. GLM and IAB did not differ significantly in ADC value, however. The MRI characteristics of these lesions are illustrated in Figures 1, 2, 3 and 4.

**Table 1:** Comparison of ADC values for mastitis subtypes

ADC Values Comparison of Subtypes	Mastitis Subtype	Case (n)	Alpha = 0.05 Subset	
			1	2
Student-Newman-Keuls test	PDM	17		1.4575208
	IAB	12	1.1053889	
	GLM	32	1.2438824	
	Significance		0.191	1.000

The AUC corresponding to ADC values between different mastitis subtypes are listed in Table 2. PDM and IAB exhibited the largest difference in ADC values (AUC = 0.794), while GLM and IAB differed least in ADC value (AUC = 0.647). Specificity was also highest (83.3%) for the comparison between PDM and IAB lesions.

**Table 2:** AUC, diagnostic threshold, and sensitivity and specificity of ADC values for mastitis subtypes

Variables (ADC Value)	AUC	Cut-off Value	Sensitivity	Specificity
PDM and IAB	0.794	1.348 × 10^-3^ mm^2^/s	0.813	0.833
PDM and GLM	0.733	1.328 × 10^-3^ mm^2^/s	0.844	0.647
IAB and GLM	0.647	1.125 × 10^-3^ mm^2^/s	0.706	0.667

The three groups did not vary significantly in non-mass internal enhancement (χ^2^ = 7.462, *P* = 0.113) or mass enhancement characteristics (shape: χ^2^ = 5.884, *P* = 0.208; margin: χ^2^ = 5.887, *P* = 0.053; internal enhancement: χ^2^ = 0.398, *P* = 0.819). These results are shown in Table 3. Likewise, there were no significant differences in lesion quadrant (*P* = 0.076), patient age (*P* = 0.445), T_2_WI signal intensity (*P* = 0.477), DWI signal intensity (*P* = 0.129), or enhancement type (*P* = 0.119) among the three groups. These results are also shown in Table 4.

**Table 3:** Lesion MR enhancement characteristics stratified by mastitis subtype (n)

	GLM	IAB	PDM	χ^2^	*P*-value
Non-mass	19	8	15		
Enhancement Distribution				16.985	0.03^*^
Focal	0	0	0		
Linear	3	0	0		
Segmental	3	0	0		
Regional	1	4	4		
Multiple regions	3	2	7		
Diffuse	9	2	4		
Internal Enhancement				7.462	0.113
Homogenous	3	0	0		
Heterogeneous	8	6	12		
Clustered Ring	8	2	3		
Mass	13	4	2		
Shape				5.884	0.208
Oval	2	1	0		
Round	1	2	0		
Irregular	10	1	2		
Margin				5.887	0.053
Circumscribed	4	4	1		
Irregular	9	0	1		
Internal Enhancement				0.398	0.819
Heterogeneous	4	1	1		
Rim Enhancement	9	3	1		

^*^ *P* < 0.05 was considered statistically significant.

**Table 4:** Patient age and lesion MR characteristics stratified by mastitis subtype (n)

	GLM	IAB	PDM	χ^2^	*P*-value
Patient Age (y)	44.47±14.68	41.33±18.79	41.34±12.71	1.617	0.445
Lesion Quadrant				11.415	0.076
Lateral-Superior	6	4	0		
Interior-Superior	9	4	2		
Lateral-Inferior	11	2	11		
Interior-Inferior	6	2	4		
T2WI Signal Intensity				3.504	0.477
Isointensity	6	0	2		
Hypointensity	2	2	2		
Hyperintensity	24	10	13		
DWI signal intensity				7.138	0.129
Isointensity	10	0	4		
Hypointensity	4	1	4		
Hyperintensity	18	11	9		

There was a significant difference in the distribution of non-mass enhancement among the three groups (χ^2^ = 16.985, *P* = 0.03, shown in Tables 3, 5). The most prevalent non-mass enhancement distribution types for PDM, GLM, and IAB were as follows: PDM = multiple-region (7/15); GLM = diffuse (9/19); and IAB = regional (4/8). Examples of distribution types for these subtypes are shown in Figures 1, 2, 3 and 4. However, Fisher’s exact test showed no significant difference in non-mass enhancement between any two groups: PDM vs. GLM (χ^2^ = 9.847, *P* = 0.210); PDM vs. IAB (χ^2^ = 7.789, *P* = 0.061); and GLM vs. IAB (χ^2^ = 1.484, *P* = 0.484). Duct ectasia appeared in 58.8% (10/17) of PDM lesions, while micro-abscesses appeared in 62.5% (20/32) of GLM lsions. These results are shown in Table 3.

**Table 5:** Enhancement types stratified by mastitis subtype (%)

Subtype	Cases	Non-Mass Enhancement	Mass Enhancement
GLM	32	19 (59.4)	13 (40.6)
IAB	12	8 (66.7)	4 (33.3)
PDM	17	15 (88.2)	2 (11.8)
Total	61	42 (68.9)	19 (31.1)

## Discussion

In this study, we compared the morphological and functional characteristics of three subtypes of non-puerperal mastitis lesions primarily using DWI and DCE MRI. ADC values extrapolated from DWI have been previously used to differentiate between abscesses and cystic tumours in various regions of the body [9, 19]. To date, however, the role of DWI in specifically differentiating between the major subtypes of non-puerperal mastitis has not been examined. Furthermore, the role of DCE MRI characteristics such as mass enhancement and non-mass enhancement in differentiating between mastitis subtypes has been limited.

Differentiating between PDM, GLM, and IAB during the non-puerperal period poses a significant diagnostic challenge due to the similar clinical presentations of these three subtypes of mastitis. Non-puerperal mastitis is commonly misdiagnosed, which can lead to incorrect treatment and a negative outcome for the patient [3, 4, 6]. Furthermore, many patients with non-puerperal mastitis present with one or several breast masses without other signs of inflammation. These masses often mimic inflammatory carcinoma both clinically and radiologically, making a definitive diagnosis of mastitis difficult [4]. It is of the utmost importance for breast specialists to both confirm the diagnosis of mastitis and determine the mastitis subtype before administering distinct therapeutic regimens, such as surgical excision, hormone therapy, or antibiotic treatment. While conventional MRI has been used as a potential imaging tool for differentiating mastitis from malignant breast lesions [7–9], it has not been effective in differentiating PDM from GLM, as PDM and GLM possess similar clinical and radiological characteristics, such as non-specific enhancement, skin thickening, diffuse oedema, and abnormal nipple configuration [8]. Additionally, IAB may mimic non-infectious mastitis on conventional MRI, especially when the clinical symptoms of IAB are atypical and the abscess has not yet formed [8, 9].

PDM, which most commonly presents in areas surrounding the areola [1, 4], is characterised by the infiltration of plasma cells and lymphocytes, and the presence of these cells is a hallmark of PDM pathologically. Previous studies [4, 12] have hypothesised that duct ectasia, followed by plasma cell-induced inflammation, plays an integral role in the pathophysiology of PDM. Lactiferous duct occlusion may lead to fluid retention and subsequent lipid spillover, thereby precipitating an influx of plasma cells and associated inflammation in the surrounding tissue. Duct ectasia was observed in 58.8% (10/17) of the PDM patients in our study. Still, PDM lesions exhibited the largest ADC value [(1.558 ± 0.34) × 10^-3^ mm^2^/s] in our study, suggesting that the inflammation associated with this mastitis subtype has, in general, less restrictive diffusion than that associated with GLM or IAB.

GLM lesions can affect any quadrant of the breast [1, 4] and have been shown to exhibit both periductal inflammation [20, 21] and the presence of microabscesses, which result in a decrease in free water diffusion as compared to healthy parenchyma [13]. In our study, microabscesses appeared in 62.5% (20/32) of GLM lesions. Minor periductal inflammation was also evident in the majority of our GLM cases, likely contributing to a significantly lower ADC value [(1.244 ± 0.32) × 10^-3^ mm^2^/s] than that for PDM [(1.558 ± 0.34) × 10^-3^ mm^2^/s]. In addition to periductal inflammation and microabscesses, the presence of blood or other viscous fluids in dilated lactiferous ducts can contribute to the restricted diffusion (and thus lower ADC value) seen in GLM lesions. Furthermore, these viscous fluids may mimic malignancies such as ductal cancers on DWI [10, 11, 16], adding to the challenge of differentiating between breast cancers and mastitis lesions.

Lastly, IAB lesions, which can be located centrally or peripherally (lesions were 50% central and 50% peripheral in our study), are characterised by the accumulation of massive inflammatory cells, microorganisms, large protein molecules, necrotic tissue, and cellular debris, all of which restrict free water diffusion [8–10]. Pathologically, squamous metaplasia of the cuboidal epithelium leads to the formation of keratin plugs, acute central inflammation, and the accumulation of cellular debris. Secondary infection ensues, leading to stagnation and subsequent abscess formation [5, 22, 23]. This pathophysiological mechanism of IAB culminates in a gross lack of free water diffusion, illustrated in our study by the hyperintensity observed on DWI and the low ADC value [(1.105 ± 0.44) × 10^-3^ mm^2^/s] as compared to PDM and GLM. While the ADC was an effective parameter for differentiating between IAB and PDM, we did not observe a significant difference in ADC value between IAB and GLM. It is possible that the similar manifestations of the microabscesses in GLM and the larger IAB lesions contributed to this lack of ADC difference, though future studies are necessary. Nevertheless, the ADC values derived from DWI have been effective in differentiating IAB lesions from cystic or necrotic tumours in various regions of the body, such as the brain and liver [9, 24–27]. Malignancies generally appear hypointense on both DWI and T_2_WI, while benign lesions such as non-puerperal mastitis show a hyperintense signal [8, 9]. Unal et al. [9] attribute this intensity difference on DWI to the different physical and biochemical contents of the lesions. Most notably, cystic and necrotic tumours contain significantly fewer inflammatory cells and cellular debris than IAB lesions, allowing for these malignancies to be differentiated from mastitis on DWI.

In sum, ADC values, which reflect the degree of water molecule diffusion through tissue, proved most effective for differentiating between mastitis subtypes in our study.

When analysing each type of lesion individually, PDM lesions exhibit the highest ADC values of the three mastitis subtypes. Furthermore, our results illustrate significant differences in ADC values among these three subtypes, as well as between members of two subtype pairs (PDM vs. GLM and PDM vs. IAB). PDM and IAB lesions differed most in ADC value, while GLM and IAB lesions differed least. We suspect that the different pathological states of PDM (duct ectasia), GLM (microabscesses), and IAB (gross restriction of free water diffusion) could be responsible for the differences in ADC values shown in Table 1. More specifically, the degree of inflammatory cell infiltration, tissue necrosis, and tissue regeneration primarily contributed to the different ADC values obtained, and aided us in our development of these diagnostic thresholds. Still, further studies on the DWI characteristics of mastitis lesions are warranted in order to obtain conclusive data.

It is known that DWI signals can be affected by T2 shine effects, while ADC values are not. In this study, we calculated mean ADC values according to the most enhanced lesion areas on DCE-MRI The ADC values were measured excluding the completely necrotic or cystic components and can reflect the characteristic of the mastitis lesions. As shown in our study, calculating ADC values using this approach can greatly aid in distinguishing the different types of non-puerperal mastitis, with the added benefits of avoiding the influences of T2 shine effects. Consistent with previous mastitis imaging studies [3, 17, 28], all mastitis lesions in our study exhibited a hyperintense signal on T2WI, with no significant difference in T2WI or DWI signal intensity among the three groups. We speculated that the fluid-like, high-protein nature of these mastitis lesions and the similarities between the microabscesses seen in GLM and the IAB lesions likely produced similar signal intensities.

Research examining the DCE MRI characteristics of mastitis has been limited [3, 4, 13]. In our study, there was no significant difference in the distribution of mass enhancement or non-mass internal enhancement among the three mastitis subtypes. We did find a significant difference in the distribution of non-mass enhancement among the three groups, though no two subtypes were significantly different from each other when compared individually. Nevertheless, we believe there is value in utilising the qualitative DCE MRI characteristics of each mastitis subtype. The most common distribution types for PDM, GLM, and IAB were multiple-region, diffuse, and regional, respectively. It is possible that these distribution types could complement the ADC values obtained from DWI to more accurately characterise the mastitis subtypes.

While our results offer promising potential for imaging various subtypes of mastitis, there are some limitations to our study. Firstly, it was performed retrospectively, and our sample size was relatively small. In addition, we did not analyse the kinetic curves of the different subtypes of mastitis. In future studies, a comparative analysis of DCE MRI versus DWI should be performed.

## Conclusions

Our results illustrate new methods of differentiating between various mastitis lesions using DWI and DCE MRI. The ADC value derived from DWI for each subtype of mastitis was especially useful for differentiating PDM from GLM and IAB lesions. This parameter has promising potential as a means of further investigating the characteristics of mastitis lesions on DWI. Additionally, the distribution of non-mass enhancement on DCE MRI offers a new lens into the imaging characteristics of mastitis. With further examination of DWI and DCE MRI in future studies, these imaging modalities could reduce the time between clinical presentation and definitive diagnosis and treatment, ultimately improving patient outcomes.

## Abbreviations

MRI: magnetic resonance imaging
DWI: diffusion-weighted imaging
DCE: dynamic contrast-enhanced
ADC: apparent diffusion coefficient
AUC: area under the receiver operating characteristic curve
